# Geranylated 4-phenylcoumarins extracted from *Mesua elegans* induced caspase-independent cell death in prostate cancer cell lines through calpain-2 and cathepsin B

**DOI:** 10.1038/s41598-020-57781-6

**Published:** 2020-01-22

**Authors:** Hani Sapili, Chai San Ho, Sharan Malagobadan, Norhafiza Mohd Arshad, Noor Hasima Nagoor

**Affiliations:** 1 Institute of Biological Science (Genetics and Molecular Biology), Faculty of Science, University of Malaya, 50603 Kuala Lumpur, Malaysia; 2 Centre for Research in Biotechnology for Agriculture (CEBAR), Faculty of Science, University of Malaya, 50603 Kuala Lumpur, Malaysia

**Keywords:** Biotechnology, Cancer, Apoptosis

## Abstract

Geranylated 4-phenylcoumarins DMDP-1 and DMDP-2 isolated from *Mesua elegans* were elucidated for their role in inducing caspase-independent programmed cell death (CI-PCD) in prostate cancer cell lines, PC-3 and DU 145, respectively. Cell homeostasis disruption was demonstrated upon treatment, as shown by the increase in calcium ion through colourimetric assay and endoplasmic reticulum (ER) stress markers GRP 78 and p-eIF2α through western blot. Subsequently, cytoplasmic death protease calpain-2 also showed increased activity during DMDP-1 & -2 treatments, while lysosomic death protease cathepsin B activity was significantly increased in PC-3 treated with DMDP-1. Flow cytometry showed a reduction in mitochondrial membrane potential in both cell lines, while western blotting showed translocation of mitochondrial death protease AIF into the cytoplasm in its truncated form. Furthermore, DMDP-1 & -2 treatments caused significant increase in superoxide level and oxidative DNA damage. Concurrent inhibition of calpain-2 and cathepsin B during the treatment showed an attenuation of cell death in both cell lines. Hence, DMDP-1 & -2 induce CI-PCD in prostate cancer cell lines through calpain-2 and cathepsin B.

## Introduction

Prostate cancer is one of the most frequently reported cancers in men worldwide with 1.8 million cases in 2018. It is commonly diagnosed in elderly men, with a high incidence rate in France, Ireland and the United States, predominantly due to the lifestyle in these developed countries^1,2^. Treatment for this cancer includes surgical removal of the prostate, radiotherapy, chemotherapy and hormone therapy. Additionally, the development of alternative medicines has increased options for treatment.

Programmed cell death (PCD), or apoptosis, is a natural process that maintains homeostasis in living organisms. While this is the most conventional cell death pathway involving the activation of caspases (CD-PCD), there is an alternative mechanism known as caspase-independent PCD (CI-PCD). CI-PCD is activated by different types of proteins or proteases depending on specific triggers and cell types^3,4^. Interestingly, many natural compounds have been reported to induce CI-PCD in different cell lines, such as berberine in colon cancer and thymoquinone in glioblastoma through activation of cathepsin B^5,6^.

Coumarins have been reported to not only have biologically important defensive functions in its plants of origin but also have anti-cancer, anti-bacterial, anti-inflammation and hepatoprotective activities in human^7,8^. However, many natural coumarins are unsuitable for therapeutic usage as they exhibit mutagenic and carcinogenic properties. As such, modification with the addition of side chains through synthetic means or extraction of its derivatives makes coumarins useful anti-cancer agents^4^. An example is geranylated phenylcoumarins, which exhibited apoptotic inducing effects on various cancer cell lines without exerting cytotoxicity on normal epithelial cell lines^9^.

In our previous study, two analogs of geranylated 4-phenylcoumarins, which are 5, 7-dihydroxy-8-(2-12methylbutanoyl)-6-[(E)]-3, 7-dimethylocta-2,6-dienyl]-4-phenyl- 2H-chromen-2-one (DMDP-1) and 5, 7-dihydroxy-8-(3-methylbutanoyl)-6-[(E)]-3, 7-dimethylocta-2,6-dienyl]-4-phenyl-2H-chromen-2-one (DMDP-2), were extracted from *Mesua elegans*, a plant commonly found in peninsular Malaysia^10^. Both analogs were tested on several cancer cell lines, namely, cervical (Ca Ski and HeLa), oral (HSC4), breast (MCF7 and MDA-MB-231), liver (HepG2), lung (A549 and SKU-L1) and prostate (PC-3 and DU 145). DMDP-1 & -2 were proven to have highest cytotoxic activity on the two different androgen independent (AI) prostate cancer cell lines, PC-3 and DU-145, respectively, but not in NP69 normal epithelial cells^11^. Further analysis found the cell death to be independent of caspases with evidence of other death proteases involvement, namely, calpain-2 and cathepsin B. Hence, this study was done to investigate the death-inducing mechanisms of CI-PCD by DMDP-1 & -2 to better understand their therapeutic potential in prostate cancers.

## Results

### Disruption of cellular homeostasis and activation of calpain-2 after treatment with both analogs

**Figure 1:**
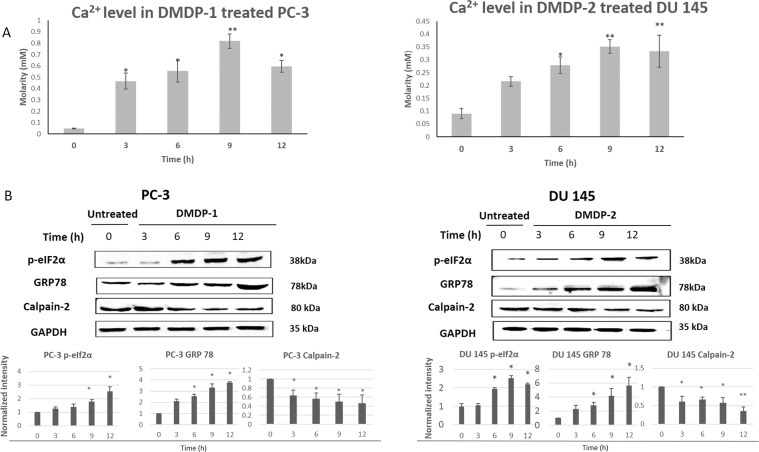
(**A**) DMDP-1 treated PC-3 and DMDP-2 treated DU 145 cells exhibited increased calcium level. The lysed untreated and treated cells were incubated with the phenolsulphonephthalein dye to stain free Ca^2+^ in blue. The colour intensity, which was proportional to the level of calcium in the lysed cells, was measured with spectrophotometer at wavelength 612 nm. Results were presented as mean normalized intensity ± S.D. of three independent experiments. (*) is used to denote p < 0.05, and (**) used to denote p < 0.005. (**B**) DMDP-1 treated PC-3 and DMDP-2 treated DU 145 cells induced ER stress and activated calpain-2. ER stress marker proteins, GRP 78 and p-eIF2α levels were measured in western blot. While Calpain-2 activity was measured by monitoring the auto-degradation of the protein through western blot. Quantification of protein bands intensities were determined by densitometry analysis and protein of interests were normalized relative to GAPDH using the image Jv1.43 software. Results were presented as mean normalized intensity ± S.D. of three independent experiments and (*) is used to denote p < 0.05.

Increase in intracellular Ca^2+^ level is important in the activation of the calcium-dependent calpain-2^12^. The Ca^2+^ levels in DMDP-1 treated PC-3 and DMDP-2 treated DU 145 were measured at 0, 3, 6, 9 and 12 hours (h) of treatment (Fig. 1A). Significant increases were observed as early as 3 h of treatment in both cell lines compared to 0 h. Abnormal increase of Ca^2+^ in the cells is often associated with the disruption in homeostasis that leads to ER stress. Therefore, to investigate whether PC-3 and DU 145 cells exhibited ER stress upon treatment with DMDP-1 & -2, the expression levels of two ER markers, p-eIF2alpha and GRP78, were monitored with western blotting at 0, 3, 6, 9 and 12 h of treatment. In DMDP-1 treated PC-3, p-eIF2alpha showed a significant increase at 9 and 12 h and DMDP-2 treated DU 145 cells at 6, 9 and 12 h. GRP78 significantly increased at 6, 9 and 12 h of treatment in comparison to 0 h in both cell lines. As such, intracellular calcium and ER stress levels are shown to increase as early as 3 to 12 h of treatment in the prostate cancer cells with both analogs (Fig. 1B).

In Suparji et. al., 2016, DMDP-1 & -2 treated PC-3 and DU 145 were investigated for the activation of calpain-2. Although calpain-2 levels changed significantly in DU 145 but not in PC-3 cells after treatment with DMDP-1 & -2, further validation experiment showed activation of calpain-2 in both cell lines when inhibited with calpeptin in the same study^11^. In this study, both PC-3 and DU 145 were treated with DMDP-1 & -2 respectively for 3, 6, 9 and 12 h and then compared with untreated cells at 0 h in a western blot, to observe the activity of calpain-2 at early hours in conjunction with the disruption in the cells cellular homeostasis. Calpain-2, which will be auto-degraded upon activation, showed a decrease in expression as early as at 3 h of treatment, and this observation was consistent up to 12 h of treatment in both cell lines (Fig. 1B), thus confirming its activity.

The same GAPDH was used for ER stress and calpain-2 analyses as protein samples used were from one treatment batch.

### Cathepsin B activity upon treatment

Besides calpain-2, lysosomal protease cathepsin B was also shown to be involved in the cell death induced in PC-3 and DU 145 cells when treated with DMDP-1 & -2 in the previous study^11^. Similarly, with cathepsin B, the treatment with CA 074 inhibited cathepsin B activity.

**Figure 2:**
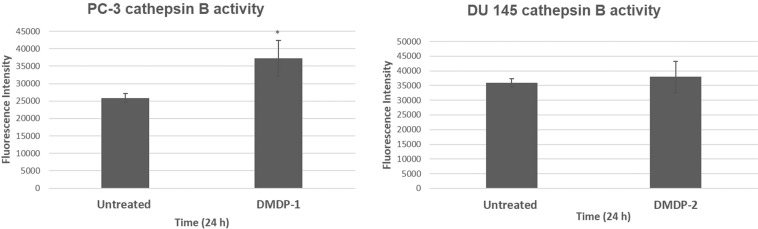
Cathepsin B activity in DMDP-1 treated PC-3 cell. Cathepsin B activity is monitored through enzyme-substrate assay kit Magic Red. Cathepsin B cleaves Magic Red substrate producing red fluorescent due to the excitation of cresyl violet fluorophore and measured with fluorescent spectro-photometer at wavelengths 592 nm excitation and 628 nm emission. Results were presented as mean intensity ± S.D. of three independent experiments and (*) is used to denote p < 0.05. Calpain-2 activated in DMDP-1 treated PC-3 and DMDP-2 treated DU 145 cells.

The activity of cathepsin B upon treatment with the analogs was monitored using an enzyme-substrate assay detection kit-Magic Red. In DMDP-1 treated PC-3 cells, a significant increase in cathepsin B activity indicated by the fluorescence intensity was observed when compared with untreated PC-3. However, in DU 145 cells treated with DMDP-2, no significant increase in fluorescence was observed in comparison with the untreated DU 145 cells (Fig. 2).

### Mitochondrial membrane potential and mitochondrial protease AIF release into the cytoplasm

**Figure 3:**
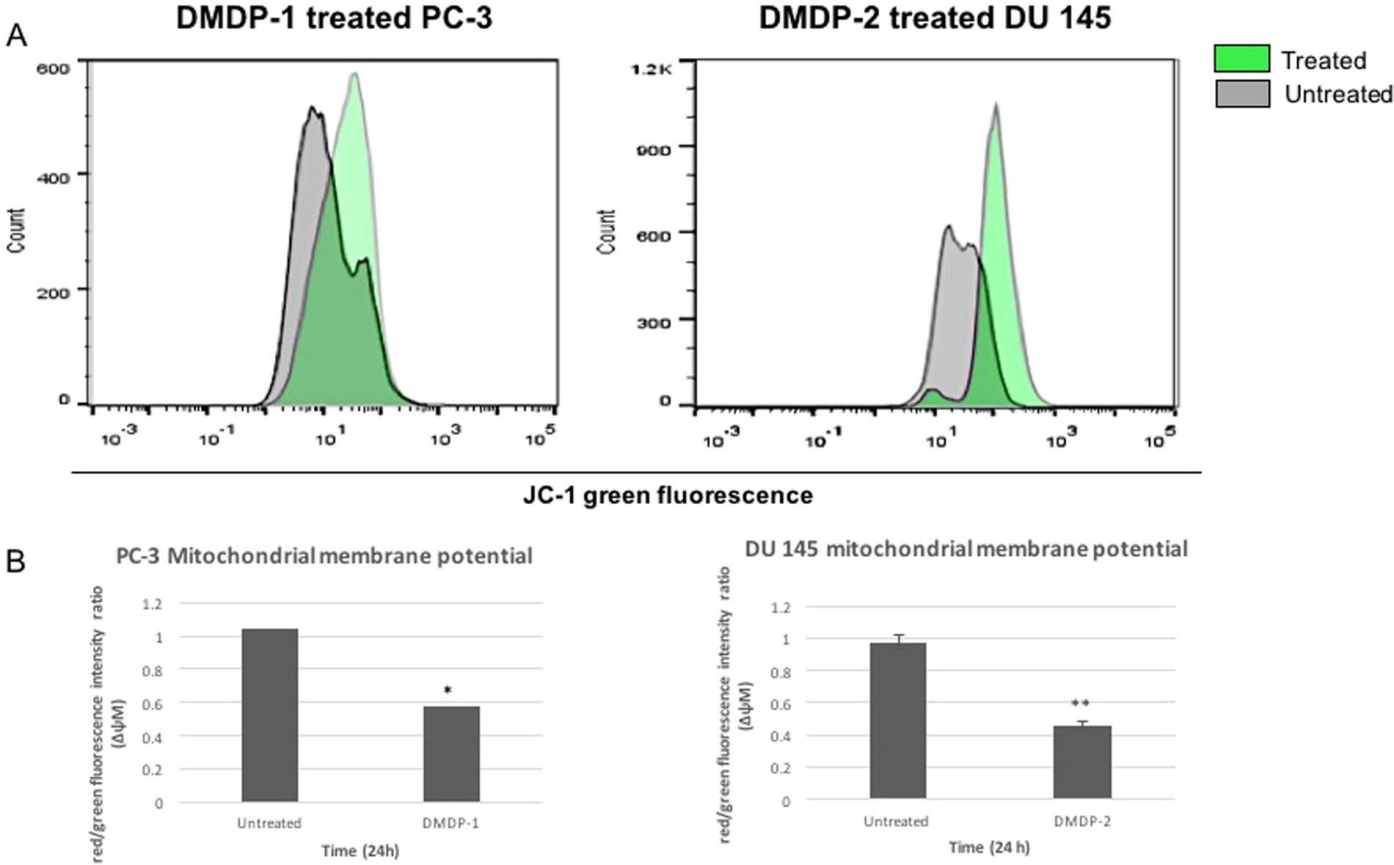
(**A**) Mitochondrial membrane potential reduced in DMDP-1 treated PC-3 and DMDP-2 treated DU 145 cells. Mitochondrial membrane potential measured with flow cytometry by JC-1 staining in cells with and without treatment for 24 hours. (**A**) Increase in green fluorescent intensity observed by the shift to the right. (**B**) ΔψM measured by calculating JC-1 red/green fluorescence intensity ratio presented in a chart. Results presented as mean intensity ± S.D. of three independent experiments and (*) is used to denote p < 0.05.

As the mitochondrial membrane permeability (MMP) is known to be affected by Ca^2+^ level changes in cells, it was important to investigate changes in the mitochondrial membrane potential in PC-3 and DU 145 cells upon treatment with DMDP-1 & -2, respectively. Mitochondrial membrane potential was measured using JC-1, a potential-dependent fluorescent stain, indicated by the emission shift from green (~529 nm) to red (~590 nm) detected with flow cytometry, where a decrease in the red/green intensity ratio is an indication of an increase in mitochondrial membrane potential, measured as ΔψM. Upon treatment, JC-1 green stained cellular count increased in PC-3 and DU 145 cells (Fig. 3A). The ΔψM of DMDP-1 treated PC-3 cells increased as indicated by the decrease in the ratio which is 0.57 ± 0.2 compared to the untreated cells at 1.03 ± 0.4. A similar result was seen in DMDP-2 treated DU 145 cells with ΔψM ratio of 0.46 ± 0.04 compared to the untreated cells at 0.98 ± 0.07 (Fig. 3B).

**Figure 4:**
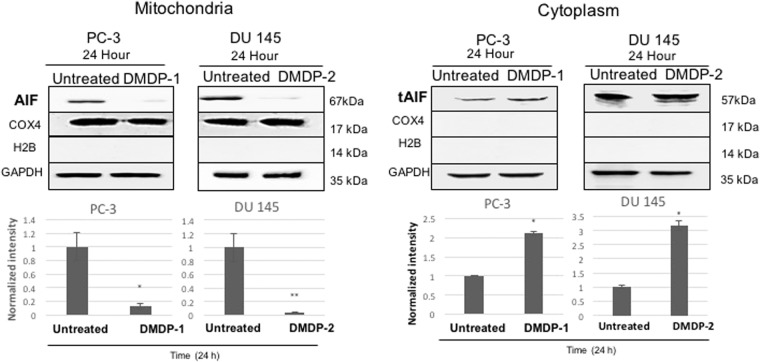
AIF released to the cytoplasm in DMDP-1 treated PC-3 and DMDP-2 treated DU 145 cells. Western blot analysis to investigate the AIF translocation between organelles in the cells. Quantification of protein bands intensities were determined by densitometry analysis and protein of interests were normalized relative to GAPDH (cytoplasm) and COX4 (mitochondrial) using the image Jv1.43 software. Results were presented as mean normalized intensity ± S.D. of three independent experiments and (*) is used to denote p < 0.05.

The protease apoptosis-inducing factor (AIF) is known to participate in the regulation of MMP. AIF is a protease which, in its mature form (62 kDa), is sequestered behind the outer mitochondrial membrane. It has the ability to induce cell death independent of caspases by translocating into the cytoplasm and subsequently into the nucleus, triggering chromatin condensation and large DNA fragmentation (50 kbp). Changes in mitochondrial membrane potential indicate disruption of the MMP. Therefore, the involvement of AIF was investigated by monitoring the translocation of the protease. The mitochondrial AIF is truncated to form a soluble AIF (tAIF, 57 kDa) to enable the translocation of tAIF into the cytoplasm and nucleus. To study the role of AIF in the cell death induced by DMDP-1 & -2 in PC-3 and DU 145 cells respectively, the expressions of AIF in mitochondria, cytoplasm and nucleus were observed through western blotting. Both PC-3 and DU 145 cells treated with their designated analogs showed a reduction in the mitochondrial 62 kDa AIF expression after 24 h of treatment. Observations of increased 57 kDa tAIF expression in the cytoplasm were made in both cell lines after treatment with the analogs (Fig. 4). However, no expression of AIF was detected in the nucleus in both cell lines.

### Increase in superoxide level

**Figure 5:**
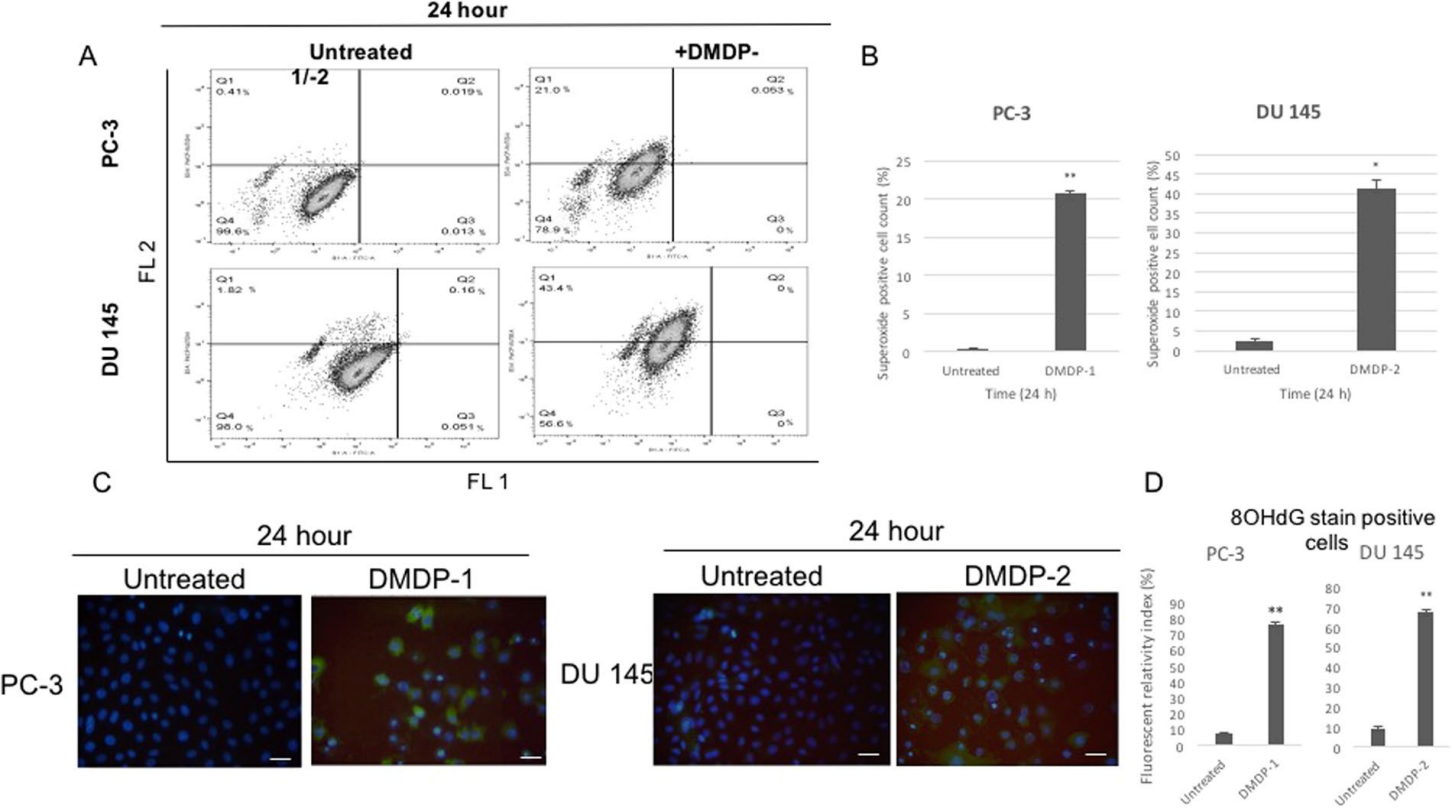
(**A**) Superoxide level increased in DMDP-1 treated PC-3 and DMDP-2 treated DU 145 cells. Cells positive of ROS superoxide (O_2_^−^) measured using fluorescent based staining assay and detected with flow cytometry, showed increase in percentage of both cell lines after 24 h of treatment (Quadrant 1). FL 1: Hydrogen peroxide (H_2_O_2_), peroxynitrite (ONOO^−^), hydroxyl radicals (HO), nitric oxide (NO) and peroxy-radical (ROO) green fluorescence stain. FL 2: Superoxide (O_2_^−^) orange fluorescence stain. (**B**) Results in the chart presented as mean intensity ± S.D. of three independent experiments. (*) is used to denote p < 0.05, and (**) used to denote p < 0.005. (**C**) DNA damage by oxidative stress induced in DMDP-1 treated PC-3 and DMDP-2 treated DU 145 cells. Immunofluorescence assay used to investigate the DNA damage caused by oxidative stress marker 8-hydroxy-2′ -deoxyguanosine (8-OHdG) in the untreated and treated cells for 24 hours. Scale bar represents 100 µm. (**D**) Chart representative of the number of cells positive with 8-OHdG green stain relative to total cell count compared with untreated cells. (*) is used to denote p < 0.05, and (**) used to denote p < 0.005.

The level of reactive oxygen species (ROS), which is reported to be involved in caspase-independent cell death mechanism, was investigated using flow cytometry. Besides AIF, calpain-2 and cathepsin B were also reported to have a role in the production of ROS. Both PC-3 and DU 145 cells, treated and untreated, were stained with a broad range of stains from the ROS detection kit (Abcam, USA) and analysed with flow cytometry. Results of DMDP-1 treated PC-3 and DMDP-2 treated DU 145 showed a significant increase in cells positive with superoxide (O_2_^−^) stain, a species of ROS, after treatment when compared to the untreated controls. Interestingly, other ROS showed no significant changes after treatment in both cell lines. (Fig. 5A,B)

### DNA damage by ROS detection

Our previous study has shown that DNA laddering, a hallmark of PCD, did not occur during treatment with both analogs^11^. Hence, DNA damage by ROS was monitored through an immunofluorescence assay detecting 8-hydroxy-oxyguanosine (8-OHdG). Both PC-3 and DU 145 showed a significant increase in cells with DNA damage as indicated by the 8-OHdG stained positive cells in comparison to the untreated cells after 24 hours of treatment (Fig. 5C,D).

### Relationship between calpain-2 and cathepsin B in the cell death induced

**Figure 6:**
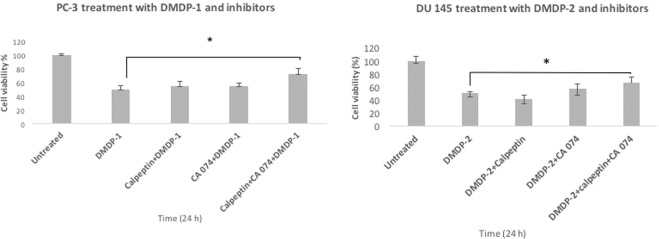
Concurrent inhibition of calpain-2 and cathepsin B attenuated cell death induced in DMDP-1 treated PC-3 and DMDP-2 treated DU 145 cells MTT cell viability assay carried out to investigate the role of calpain-2 and cathepsin B in CI-PCD induction using their respective inhibitors, calpeptin and CA-074. PC-3 was treated with DMDP-1 and DU 145 with DMDP-2, co-cultured with either calpeptin or CA-074 or both inhibitors combined. Comparison made with uninhibited cells 24 h of treatment presented as mean intensity ± S.D. of three independent experiments and (*) is used to denote p < 0.05.

Activities of calpain-2 and cathepsin B during treatment with DMDP-1 & -2 were inhibited, and cell viability was assessed using the MTT assay. The aim to inhibit calpain-2 and cathepsin B activities was to investigate the roles they play in the induced CI-PCD. Calpain-2 activity was inhibited with calpeptin while cathepsin B activity was inhibited with CA-074. It was observed when inhibited and with 24 h of treatment with the two analogs, cell viability of PC-3 was 72.1% (p-value = 0.04) and DU 145 was 67.2% (p-value = 0.02), compared to the uninhibited control (Fig. 6). The results from this experiment indicated that the inhibitors of the two proteases, calpeptin and CA074, were able to increase cell viability and attenuate cell death, confirming induction of cell death by calpain-2 and cathepsin B when treated with DMDP-1 & -2.

## Discussion

In this study, ER stress and calcium overload were observed during early treatment of DMDP-1 & -2 in PC-3 and DU 145 cells, respectively. ER is responsible for protein translocation, folding and post-translation modification, and also functions as calcium storage in non-muscle cells. ER membranes contain many Ca^2+^ binding proteins and channels regulating the balance and movement of Ca^2+^ throughout the cells^13–15^. As such, disturbance in the balance within the cellular environment by pathological or physiological insults will eventually lead to ER stress^13^. This condition causes an overflow of Ca^2+^ into the cytoplasm, causing disruption in the ER functions and inducing cytotoxicity in cells^4^. However, there is an ongoing debate as to whether Ca^2+^ cause ER stress in the cells or vice versa^14^. Evidence from various studies have shown that both ER stress and Ca^2+^ accumulation in the cells often occur at a proximal time to each other^15,16^. Nevertheless, in the respective prostate cancer cell lines, treatment with DMDP-1 & -2 induced disruption of Ca^2+^ balance and triggered ER stress in the cells.

Calpain-2 is a cytoplasmic cysteine protease that is activated by a micromolar concentration of intracellular Ca^2+^. Activated calpain-2 will be auto-degraded before carrying out proteolysis of its substrates^17^. ER stress and calcium overload in the cells have shown to provide a favorable environment for calpain-2 activation^3,18^. In our study, activation of calpain-2 was detected in parallel to the increase in Ca^2+^ and ER stress level, which occurred immediately after treatment with the analogs.

Cathepsin B is a lysosomic protease that is sensitive to the changes in intracellular Ca^2+^. Increase in Ca^2+^ leads to the lysosomal membrane permeabilisation (LMP) enabling release of cathepsin B into the cytosol, where it has been reported to induce both CD and CI-PCD. In this study, treatment of DMDP-1 in PC-3 induced cathepsin B activity but not in DMPD-2 treated DU 145 cells, which can be attributed to the high baseline of cathepsin B expression and activity in DU 145 cells^19^.

The ability of both calpain-2 and cathepsin B in inducing cell death is seen not only in CD-PCD but also in CI-PCD, making these two proteases potential targets for drug development in cancer therapy. Interestingly, recent findings have shown that calpain-2 and cathepsin B were observed to work together to mediate cell death in an event described as calpain-2 and cathepsin B axis^20^. Our study has shown that inhibition of both calpain-2 and cathepsin activity concurrently with treatment of DMDP-1 & -2 was able to attenuate significant cell death in PC-3 and DU 145 cell lines, suggesting that both proteases are required in the DMDP-1& -2 induced CI-PCD.

Besides ER, mitochondrion is another organelle that requires Ca^2+^ for many of its functions, especially in the mitochondrial membrane transport and permeability^4^. Mitochondrial permeability transition (MPT) is one of the mechanisms in mitochondrial transport that is highly regulated by the Ca^2+^ and oxidative stress levels in the cells. Exposure to the high level of both Ca^2+^ and oxidative stress will trigger the formation of MPT pores. High MPT will eventually increase the mitochondrial membrane potential and lead to mitochondrial membrane depolarization, which will allow an uninhibited flow of ions and proteins across the membrane^21,22^. The reduction in mitochondrial membrane potential observed in the cells upon treatment indicated a disruption of the membrane integrity, which could explain the release of AIF from the mitochondria into the cytoplasm.

Truncated AIF released from the mitochondria conventionally will be released to the cytoplasm and further translocated to the nucleus to induce large DNA fragmentation^23^. However, in this study, no AIF was observed in the nucleus following the release into the cytoplasm. Next, the superoxide level in the prostate cancer cell lines upon treatment with the analogs was investigated, as AIF is known to have redox activity^24^. The superoxide (O_2_^−^) level was higher upon treatment, which causes oxidative stress. This was supported by the immunofluorescence assay, which showed an increase in 8-OHdG, a byproduct of oxidative DNA damage used to evaluate DNA damage caused by endogenous oxidative stress.

## Conclusion

As a conclusion, DMDP-1 & -2 treatment in PC-3 and DU 145 cell lines respectively have shown to induce CI-PCD and disrupt cellular homeostasis by increasing Ca^2+^ and ER stress level, activating known death proteins calpain-2 and cathepsin B, translocating mitochondrial AIF to the cytoplasm and elevating superoxide level to the point of inducing oxidative DNA damage. Thus DMDP-1 & -2 isolated from *Mesua elegans* are potentially important anti-cancer agents in prostate cancer.

## Material and Methods

### Plant materials

The bark of *Mesua elegans* (King) Kosterm was collected from Sungai Badak Forest Reserve, Kedah, Malaysia. The sample was identified by Mr. Teo Leong Eng and deposited in the Department of Chemistry, Faculty of Science, University of Malaya herbarium (Ref. No: KL5232). Geranylated 4-phenylcoumarin analogs DMDP-1 and DMDP-2 were extracted and with ≥98% purity (Supplementary Fig. 1) from the bark using high performance liquid chromatography by Mr. Fadzli Bin Md Din, Department of Chemistry, Faculty of Science, University of Malaya.

### Pharmacological inhibitors

Calpain-2 inhibitor, calpeptin and cathepsin B activity inhibitor ≥99%, CA-074 were purchased from Merck (Germany).

### Cell culture

The prostate cancer cell lines, PC-3 and DU 145 were purchased from American Type Culture Collection (ATCC) (Manassas, VA, USA). Both cells were cultured in 10% (v/v) fetal bovine serum and 1% penicillin/streptomycin supplemented RPMI 1640. Cells were cultured at 37 °C with 5% CO_2_/95% air as monolayers.

### Cell treatment

PC-3 cells were treated with DMDP-1 at IC50 13 μM, while DU 145 cells were treated at IC50 5 μM detected through MTT assay (Supplementary Fig. 2).

### Protein extraction

All untreated and DMDP-1&-2 treated PC-3 and DU 145 cells were harvested with trypsinisation and centrifuged at 400 × g for 5 minutes prior to extraction.

### Cytoplasmic protein extraction

The cytoplasmic proteins were extracted from the cells following the protocol from the NE-PER^1^ Nuclear and Cytoplasmic Extraction Kit (Thermo Fisher Scientific, USA).

### Mitochondrial protein extraction

The mitochondrial protein fractions were prepared using the Mitochondrial Isolation Kit for Cultured Cells (Thermo Fisher Scientific, USA).

### Nuclear protein extraction

The nuclear proteins were extracted following the two-minute cell fractionation method-REAP^25^. Harvested cells were counted to standardise the cell numbers of each samples before being lysed in 0.1% NP40 alternative (Calbiochem, USA) in PBS for 30 seconds. Subsequently the lysed cells were centrifuged at top speed for 10 seconds. The pellets collected were resuspended again in 0.1% NP40 alternative for 30 seconds and centrifuged at top speed for another 10 seconds to get the final pellets of the nuclear fractions.

### Western blot

The protein concentrations were measured with a spectrophotometer at 562 nm wavelength using Pierce^TM^ BSA Protein Assay Kit (Thermo Fisher Scientific, USA). Equivalent amount of proteins were loaded onto SDS-polyacrylamide gel for protein separation before being transferred onto nitrocellulose membranes. Immunoblotting was done by incubating the membranes with primary antibody overnight at 4 °C followed by incubation with horseradish peroxidase (HRP)-linked secondary antibody. A total of 8 primary antibodies were used against calpain-2, cathepsin B, GRP-78/Bip, p-eIF2 alpha, apoptotic inducing factor (AIF), GAPDH, H2B and COX IV from Cell Signaling Technology, Danvers, MA. Protein bands were detected through chemiluminescence by subjecting the membrane to WesternBright Quantum (Advansta, USA) prior to visualisation with a chemiluminescent imaging system (Fusion FX7). GAPDH, Cox IV and H2B were used for normalization of band intensity for cytoplasmic, mitochondrial and nuclear fractions respectively by using a densitometry sofware imageJ v1.48 (NIH, USA).

### Intracellular calcium measurement

A total of 4 × 10^6^ untreated and DMDP-1 & -2 treated PC-3 and DU 145 cells were harvested with trypsinisation and centrifuged at 400 g for 5 minutes. The cell lysis and measurement of the intracellular calcium concentration were done as recommended following the protocol from the calcium measurement kit QuantiChromeTM Calcium Assay Kit (BioAssay, USA).

### Immunofluorescence assay

Both PC-3 and DU 145 cells were plated on 24-well plates and treated with the analogs at their respective IC_50_ values. Cells were fixed with 4% formaldehyde-PBS, rinsed with PBS before being incubated in a blocking buffer of 1× PBS/5% normal serum/0.3% Triton™ X-100 for 1 hour in room temperature. Blocking buffer was removed and incubated with primary antibodies: mouse monoclonal antibody against 8-hydroxy-oxyguanosine (8-OHdG) (Santa Cruz Biotechnology, USA) at 4 °C overnight. Subsequently, the cells were rinsed with PBS and incubated with secondary antibodies of Goat anti-Mouse IgG H&L (Alexa Fluor® 488) (Abcam, USA) for one hour in room temperature.

### Cathepsin B activity measurement

All untreated and DMDP-1&-2 treated PC-3 and DU 145 cells were harvested with trypsinisation and centrifuged at 400 × g for 5 minutes prior to measurement. Cathepsin B activity was measured following the protocol from a cathepsin B activity measurement kit Magic Red® Cathepsin assay kit. In brief, harvested cells were incubated with a fluorescent staining solution Magic Re® for one hour at 37 °C protected from light and the fluorescence intensity was measured by microplate reader at 592 nm and 628 nm excitation and emission wavelength respectively.

### Reactive oxygen species detection

Untreated and DMDP-1&-2 treated PC-3 and DU 145 cells were harvested with trypsinisation and centrifuged at 400 × g for 5 minutes at room temperature prior to analysis. Reactive oxygen species detection (ROS) was done following the protocol of Cellular ROS/Superoxide Detection Assay Kit (Abcam, USA). Harvested cells were rinsed with wash buffer before being resuspended in ROS/Superoxide Detection solution containing the fluorescent dyes and incubated in the dark for 30 minutes at room temperature. Total cells of 20 000 were measured for the fluorescent products generated using flow cytometer equipped with a blue laser (488 nm filter).

### Statistical analysis

All results were expressed as mean ± S.D of data obtained from at least three independent bio- logical replicates. One-way ANOVA was employed to assess the significant differences between controls and treated samples, with a p-value < 0.05 considered to be significant.

## Supplementary information

Supplementary Dataset 1.

## Data Availability

All relevant data are within the paper and its Supporting Information files.
