# Supplementary material for: Geranylated 4-phenylcoumarins extracted from Mesua elegans induced caspase-independent cell death in prostate cancer cell lines through calpain-2 and cathepsin B
Source: Sci Rep. 2020 Jan 22;10:986. doi: 10.1038/s41598-020-57781-6 (PMC6976669; doi:10.1038/s41598-020-57781-6)
Supplement: Supplementary file 1 — Supplementary Dataset 1. [file 41598_2020_57781_MOESM1_ESM.pdf]

Title

Geranylated 4-phenylcoumarins extracted from *Mesua elegans* induced caspase-independent cell death in prostate cancer cell lines through calpain-2 and cathepsin B

Authors

Hani Sapili[1], Chai San Ho[2], Sharan Malagobadan[2], Norhafiza Mohd Arshad[2] & Noor Hasima Nagoor[1,2]

Affiliations

1. Institute of Biological Science (Genetics and Molecular Biology), Faculty of Science, University of Malaya, 50603, Kuala Lumpur, Malaysia.

2. Centre for Research in Biotechnology for Agriculture (CEBAR), Faculty of Science, University of Malaya, 50603, Kuala Lumpur, Malaysia.

Corresponding Author

Noor Hasima Nagoor
hasima@um.edu.my

Supplementary Figure 1

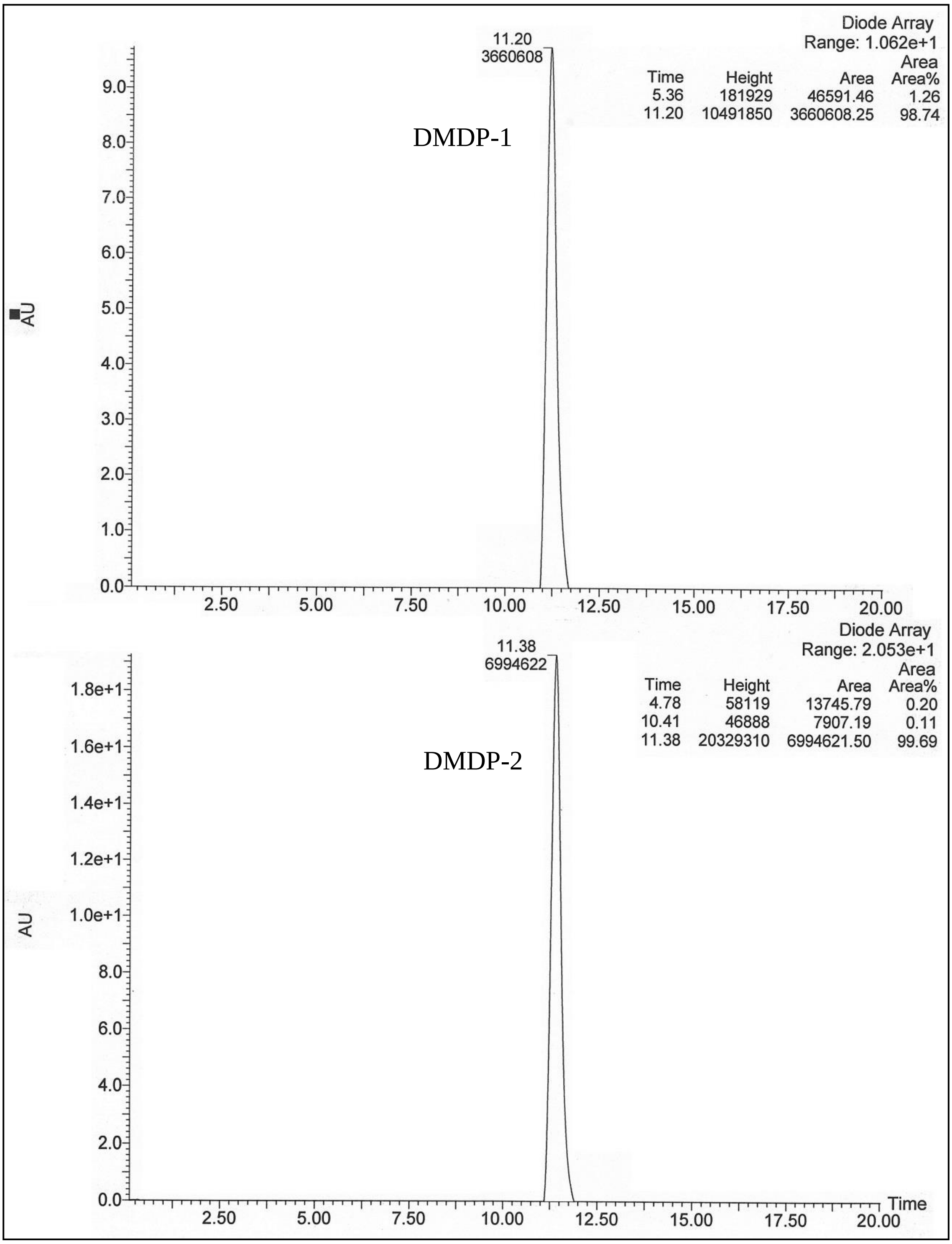

Chromatogram of purity percentage for DMDP-1 & -2 as obtained from HPLC analysis. Condition: column, ZORBAX Eclipse Plus C18, 4.6 mm i.d. x 150 mm x 3.5 µm; mobile phase, two solvents: A, 0.1% formic acid in H_20 and B, 0.1% formic acid in MeOH; the elution program at 0.6 mL/min as isocratic with 95% B (0–20 min).

Supplementary Figure 2

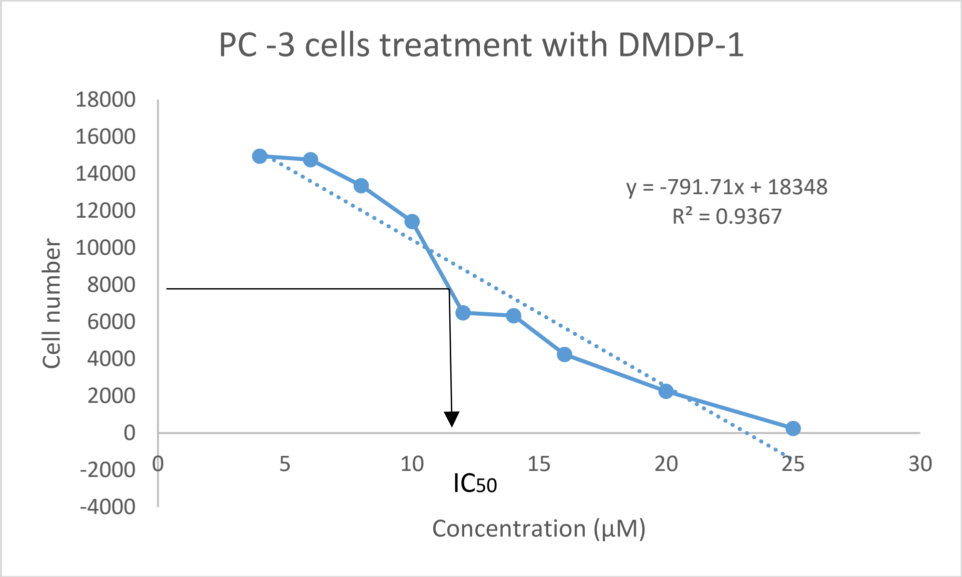

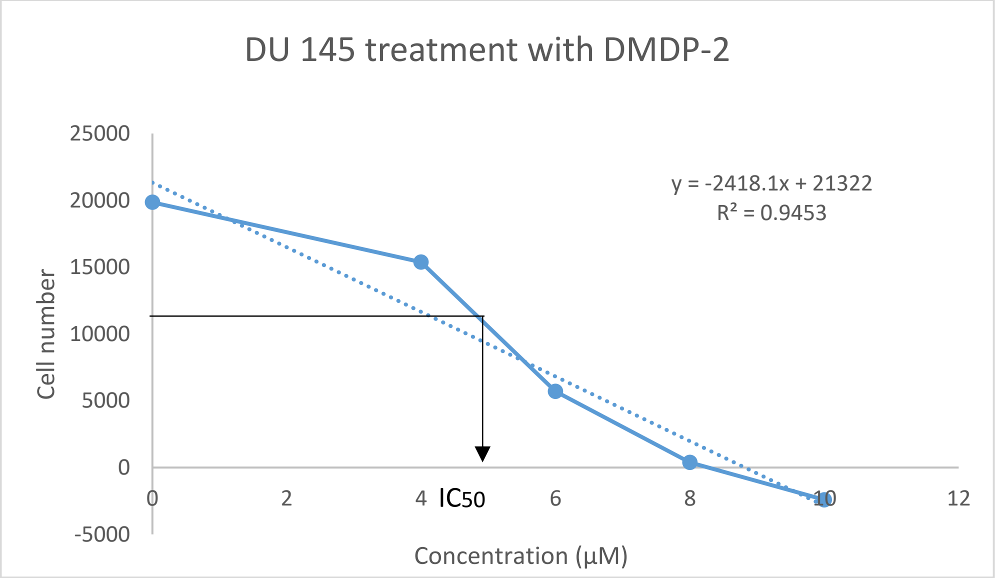

Viability (%) = [100% – cytotoxicity (%)]; where cytotoxicity (%) = [(absorbance value of solvent - absorbance value of DMDP-1/-2 treated cells)/absorbance value of untreated cells] × 100%. IC_{50} values for DMDP-1 & -2 were determined from the graph at 50% cell viability.

IC_{50} DMDP-1 = 13 µM
IC_{50} DMDP-2 = 5 µM

Supplementary Info: Full-length blots from Results: Figure 1B and Figure 4

Figure 1B

PC-3 p-eIF2α, 12% gel

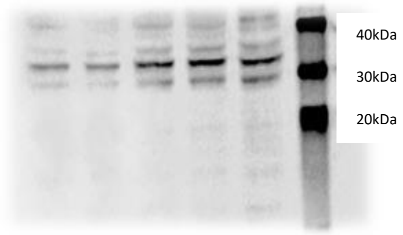

PC-3 GRP 78, 8% gel

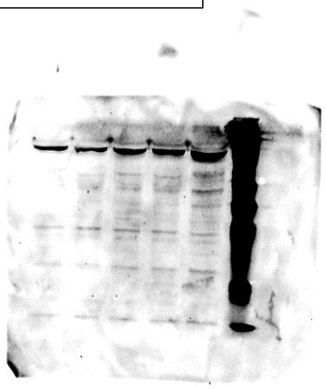

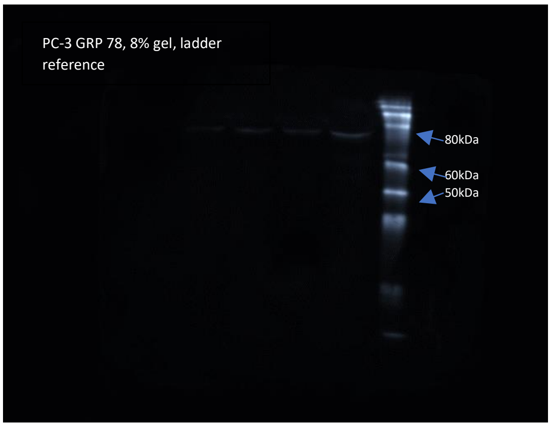

PC-3 calpain 2, 8% gel

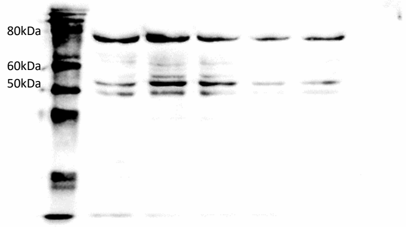

PC-3 GAPDH, 8% gel

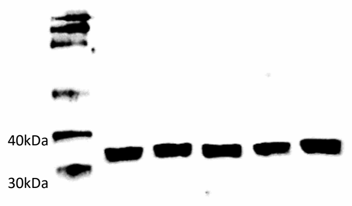

DU 145 p-eIF2α, 12% gel

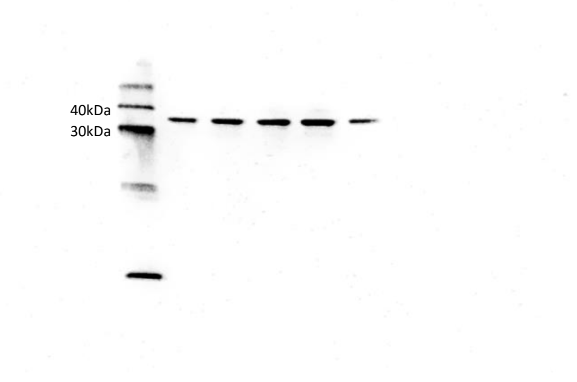

DU 145 GRP78, 8% gel. Stained with the same monoclonal GRP78 antibody used for PC-3

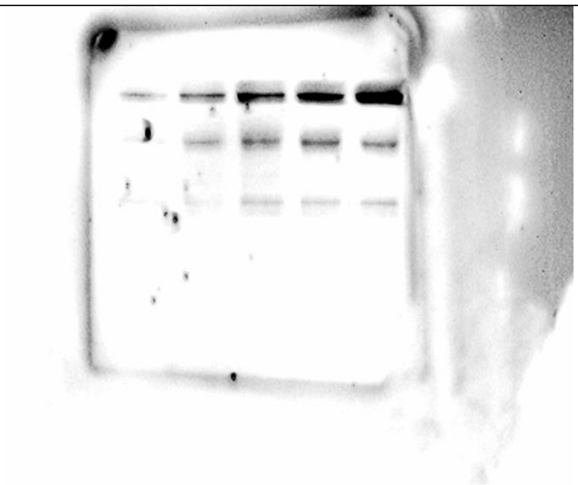

Calpian-2, 8% gel

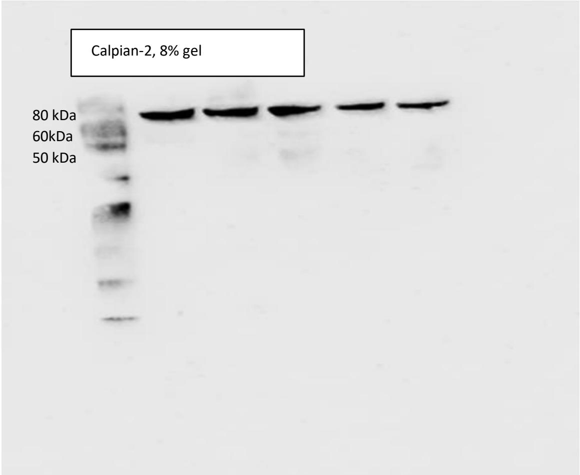

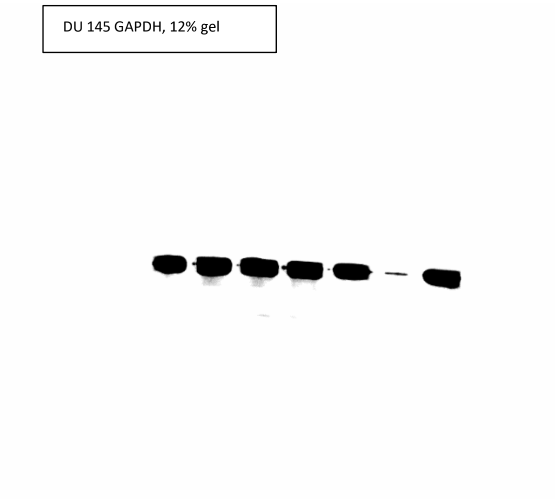
DU 145 GAPDH, 12% gel

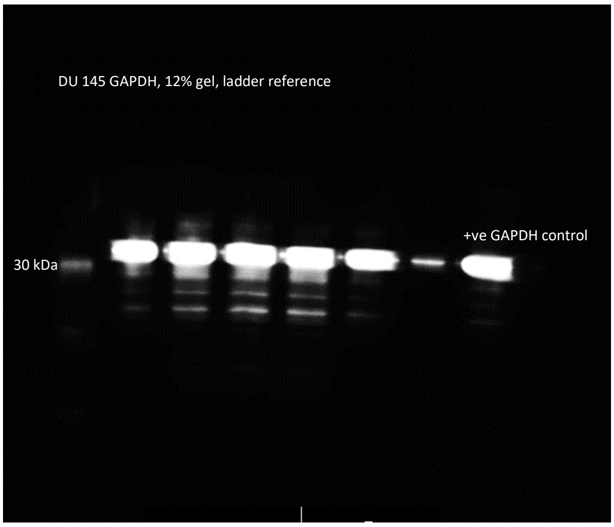
DU 145 GAPDH, 12% gel, ladder reference
+ve GAPDH control
30 kDa

Figure 4

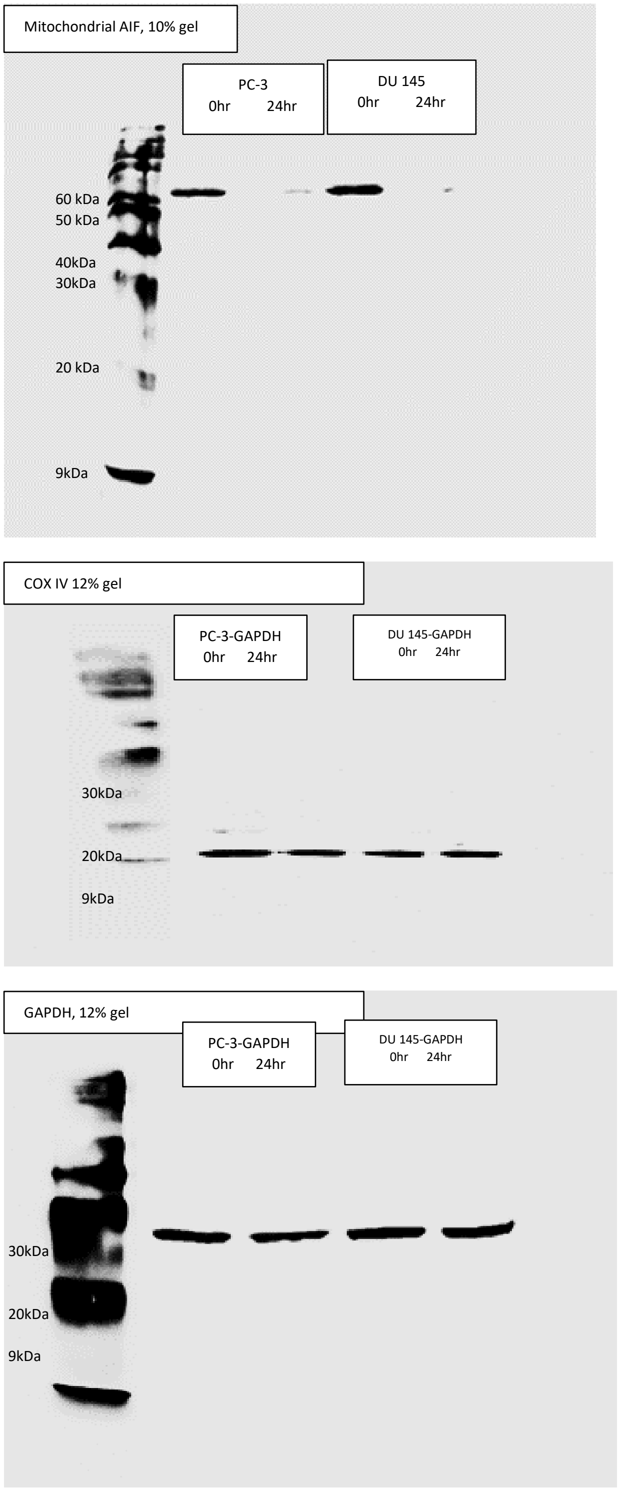
Mitochondrial AIF, 10% gel
PC-3
0hr 24hr
DU 145
0hr 24hr
60 kDa
50 kDa
40kDa
30kDa
20 kDa
9kDa
COX IV 12% gel
PC-3-GAPDH
0hr 24hr
DU 145-GAPDH
0hr 24hr
30kDa
20kDa
9kDa
GAPDH, 12% gel
PC-3-GAPDH
0hr 24hr
DU 145-GAPDH
0hr 24hr
30kDa
20kDa
9kDa

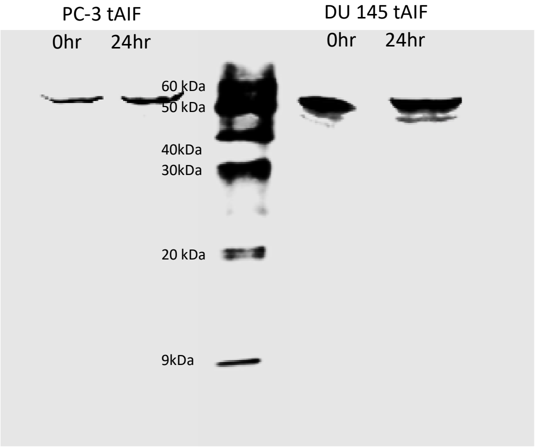
PC-3 tAIF
0hr
24hr
DU 145 tAIF
0hr
24hr
60 kDa
50 kDa
40kDa
30kDa
20 kDa
9kDa

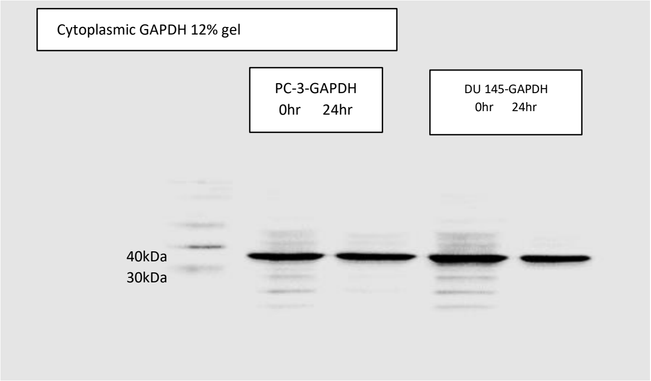
Cytoplasmic GAPDH 12% gel
PC-3-GAPDH
0hr
24hr
DU 145-GAPDH
0hr
24hr
40kDa
30kDa
